# “It's the Exact Same Treatment … Except Only 5 Min Away”: A RE‐AIM Evaluation of a Speech‐Language Pathology Shared‐Care Model for Rural Patients With Head and Neck Cancer

**DOI:** 10.1002/hed.70311

**Published:** 2026-05-12

**Authors:** Corey Patterson, Elizabeth C. Ward, Rebecca L. Packer, Jasmine Foley, Laurelie R. Wishart, Clare L. Burns, Sally Pether, Wendy Comben

**Affiliations:** ^1^ Speech Pathology Department The Townsville University Hospital, The Townsville Hospital and Health Service, Queensland Health Townsville Australia; ^2^ Centre of Functioning and Health Research, Metro South Hospital and Health Service, Queensland Health Brisbane Australia; ^3^ School of Health and Rehabilitation Sciences The University of Queensland Brisbane Queensland Australia; ^4^ School of Medicine and Dentistry Griffith University Brisbane Australia; ^5^ Office of the Chief Allied Health Practitioner, Metro North Hospital and Health Service, Queensland Health Brisbane Australia; ^6^ Speech Pathology and Audiology Department The Royal Brisbane and Women's Hospital, Metro North Hospital and Health Service, Queensland Health Brisbane Australia; ^7^ Speech Pathology, The Mount Isa Hospital, North West Hospital and Health Service, Queensland Health Mount Isa Australia

**Keywords:** head and neck cancer, implementation, rural, shared‐care, speech language pathology

## Abstract

**Background:**

Challenges accessing post‐treatment speech‐language pathology (SLP) services can add to treatment burden for rural patients with head and neck cancer (HNC). Hence, the aim of this study was to evaluate a shared‐care SLP service designed to support local SLP access for patients with HNC, using the RE‐AIM framework.

**Methods:**

RE‐AIM evaluation involved 5 staff and 23 patients. Data sources included service statistics, costs, quality of life measures, satisfaction surveys, and interviews.

**Results:**

Shared‐care demonstrated strong reach and effectiveness: 91% of patients received most or all care locally. Average patient‐attributable cost savings were AUD$801.94/person. Patients were highly satisfied, reporting equivalent care between local and tertiary services. Clinicians valued professional development and service integration. Communication systems and infrastructure assisted implementation. Policy alignment supported maintenance.

**Conclusion:**

The shared‐care model enhanced rural SLP service access while maintaining quality care and lowering patient burden. Effective communication and clinical support were key enablers, demonstrating shared‐care as a sustainable approach for rural post‐HNC treatment SLP services.

## 1 Introduction

Recovery following head and neck cancer (HNC) treatment is frequently characterized by persistent side effects that compromise communication and swallowing, often continuing well into the post‐acute phase [1]. Dysphagia, xerostomia, mucositis, and speech and voice changes are among the most common and persistent complications, particularly for patients who receive chemoradiotherapy [1, 2]. These impairments significantly affect patients' functional independence and psychosocial well‐being and can add to financial burden [3, 4, 5, 6]. Adequate speech‐language pathology (SLP) support during the early survivorship period is therefore essential for monitoring and managing these post‐treatment impacts [7, 8]. Hence, clinical guidelines and workforce strategies have long recognized the importance of timely access to SLP services during this critical period [9, 10, 11].

Despite this recognized need, access to ongoing SLP management following HNC treatment is not equally distributed across the Australian healthcare landscape. Centralisation of specialist services in large metropolitan tertiary centres creates significant disparities for patients living in regional, rural, and remote areas [4, 12, 13]. These patients are often required to travel long distances to access SLP follow‐up, despite some having needs that could be managed locally with adequate clinical support. Although professional guidelines indicate that all SLPs should be able to manage non‐complex dysphagia and communication issues [7], the ability to deliver competent HNC care depends heavily on clinicians' experience, training, and access to specialist mentoring. In rural contexts, local SLP clinicians may be isolated from peer support or continuing education, and as a result, many feel under‐prepared to manage HNC caseloads [14]. This contributes to an ongoing pattern in which patients with HNC are transferred back to tertiary services, even for manageable care needs, exacerbating their care burden and perpetuating inefficiencies in healthcare services [12, 15, 16]. Considering these challenges, there is increasing interest in innovative models of care that could enable rural patients to access safe and effective services closer to home [6].

Telehealth is recognized as a valuable strategy for mitigating geographic barriers and has demonstrated feasibility in supporting HNC‐related SLP care [17]. Telehealth can help reduce patient travel burden and allow regional and rural patients to access specialist cancer services as needed. However, it is not a universal solution. Some rural and remote patients still experience a range of barriers to telehealth access, including inadequate internet coverage in remote areas, a lack of suitable telehealth platforms (e.g., access to a smartphone or computer), and low technology/computer literacy [18]. There is also a proportion of patients who still prefer in‐person care over virtual support. Furthermore, the exclusive reliance on telehealth services delivered by tertiary cancer care clinicians only risks perpetuating centralized models of expertise, limiting opportunities for rural clinician skill development and reducing the integration of care within local health systems.

Shared‐care represents an alternative model for patients to receive care closer to home, grounded in a philosophy of distributed expertise and collaborative management between specialist tertiary teams and local clinicians. In SLP services for HNC, shared‐care offers rural clinicians the potential to deliver day‐to‐day care while still drawing on support from tertiary‐based colleagues for complex or evolving patient needs. It supports patient‐centered care through tangible benefits such as improved access to services, reduced travel, and lower costs [19], builds workforce capability, and streamlines care, particularly when combined with telehealth and access to a common electronic medical record (EMR) [20, 21, 22]. The benefits of shared‐care, though, are not solely logistical: empowering rural clinicians to work at full scope can also strengthen job satisfaction, retention, and local system resilience [23, 24].

Despite conceptual support of shared‐care models, to date, there has been limited published evidence evaluating the implementation of SLP shared‐care models in the rural HNC context. Existing literature on shared‐care has largely focused on feasibility or patient satisfaction [19, 20, 25, 26], with insufficient examination of patient outcomes, implementation processes, workforce impact, and sustainability within real‐world public health networks. This represents a critical gap in knowledge of the implementation of shared models of care, particularly given the increasing prevalence of HNC survivorship [27] and the well‐documented disparities in rural health service access [28, 29].

The shared‐care model evaluated in this study was first described as a pilot SLP service [30]. Developed through an action research methodology and informed by a series of studies exploring issues with SLP service access within a cancer service network [12, 13, 31], this SLP HNC shared‐care model was initially tested with 8 patients. Findings revealed successful transfer of a proportion of SLP services to local care. Participants were highly satisfied with the support they received from local services, and local clinicians reported increased HNC skills [30]. Whilst preliminary data confirmed the initial model's feasibility, it was acknowledged that a more rigorous evaluation of the implementation of the model was needed.

At the time of the current study, the shared‐care model described by Foley et al. [30] had been operating for almost 3 years. The aim of this research was to utilize a systematic implementation evaluation framework to guide a formal evaluation of the shared‐care SLP HNC service model. The objective was to determine the model's success in delivering its key objective of supporting access to SLP services closer to home for rural patients with HNC. Specifically, the study sought to examine patient outcomes, service delivery processes, clinician experiences, and the feasibility of maintaining the model within a public health network. The current evaluation builds upon existing research that documents the needs and experiences of rural HNC patients and clinicians accessing SLP services within this health network [12, 13, 31] and responds to broader calls for innovative, sustainable models to address rural health workforce and service access challenges [6, 32].

## 2 Methods

This implementation evaluation was guided by the RE‐AIM (Reach, Effectiveness, Adoption, Implementation, Maintenance) framework, which provides a structured approach to examining both clinical and organizational dimensions of interventions [33]. RE‐AIM explores how an intervention affects patient access and outcomes, how clinicians and organizations adopt it, and whether it can be maintained in routine practice over time [34, 35]. The RE‐AIM framework has been widely applied to integrated care models, demonstrating its utility in capturing factors that support success and implementation challenges [36].

The prospective RE‐AIM analysis of the SLP HNC shared‐care service model in this research involved the study of all consecutive eligible adult participants from rural areas eligible for shared‐care over a 10‐month period, from August 2023 to May 2024, at which time the model had been in use within the health service for almost 3 years. Analysis involved all RE‐AIM dimensions to ensure the model was evaluated broadly and from multiple perspectives, including costs [34]. The project received ethical clearance from Townsville Hospital and Health Service Human Research Ethics Committee (HREC/18/QTHS/159). All participants provided informed consent.

### 2.1 Clinical Context

The SLP HNC shared‐care model is delivered by a network of services within a public health system in Australia. This network consists of one inner regional tertiary cancer centre and four smaller facilities located 90–900 km from the tertiary facility, which will be collectively referred to as the “rural” or “local” services. Details of this network have been reported in previous publications [12, 13, 30]. The tertiary site offers a dedicated multidisciplinary cancer care service, providing definitive HNC treatment and specialist SLP services. The rural SLP services provide care for patients within their local catchment. Across the rural sites, the number of speech‐language pathologists employed ranges from 0.6 to 2 full‐time equivalent staff.

### 2.2 The SLP HNC Shared‐Care Model

The shared‐care model evolved from research identifying significant barriers to rural patients' access to post‐HNC treatment SLP services [12, 13, 31]. Then, based on a successful pilot trial [30], the model was subsequently implemented as standard practice across the network.

Prior to the implementation of the shared‐care model (i.e., the historical model), the rural SLP sites offered limited services to patients who had undergone HNC treatment, and most rural patients were required to return to the tertiary centre for most, if not all, ongoing SLP care once they had finished cancer treatment. In contrast, the current SLP HNC shared‐care model enables rural HNC patients to receive their post‐treatment SLP care (i.e., ongoing SLP services provided once definitive oncology treatment is complete) closer to home through coordinated care between local rural and tertiary SLP services. Suitable patients, defined as those requiring SLP services that can be delivered within local service capability, are identified as they commence oncology treatment at the tertiary centre. At that time, the speech‐language pathologist at the tertiary centre alerts the rural clinician to treatment plans and the date of patients' estimated return home. This early notification enables rural clinicians to complete any necessary upskilling and schedule local appointments. In the final week/s of treatment, clinicians from the local and tertiary sites meet via telehealth to discuss ongoing care needs, ideally with the patient in attendance to introduce the local SLP team.

Upon completion of oncology treatment, the priority is to ensure ongoing SLP care is delivered locally. The exceptions are when (1) specialized services are needed that are only available at the tertiary centre (e.g., a videofluoroscopic swallow study), (2) patients choose to align their SLP sessions with other appointments occurring at the tertiary center (e.g., choosing to see the tertiary speech‐language pathologist on the same day as attending an oncology appointment), or (3) when clinical issues exceed local clinician capabilities. When this occurs, either joint telehealth sessions involving both the local and tertiary speech‐language pathologists, and/or the patient are conducted for mentoring and troubleshooting, or the patient returns to the tertiary site for care.

The model maintains shared‐care practice through regular communication between sites, facilitated by a common EMR and telehealth sessions. Local services may offer facility‐based appointments, home visits, or telehealth sessions, depending on patient needs. To support quality of service, rural clinicians' capabilities are regularly reviewed by the tertiary centre clinician using the Speech Pathology Cancer Care Framework (Adults) [10], which outlines 22 clinical assessment and 11 intervention tasks. This framework helps identify local capability and training needs. Rural clinicians also have access to an online learning platform with a resource repository and mentoring by the tertiary clinician via telehealth sessions or tertiary site observation visits. Regular contact between the tertiary and local clinicians is encouraged to optimize patient care and maintain effective working relationships.

### 2.3 Participants

There were two participant cohorts recruited to this service evaluation: (a) consecutive adult patients eligible to receive shared‐care, and (b) SLP staff involved in the delivery of the service at each site. Eligible patients were individuals aged 18 years and above with HNC who received curative intent treatment at the tertiary cancer centre within the 10‐month recruitment period, lived within the catchment areas of the 4 rural sites, and were predicted to require SLP services post‐oncology management. Patients receiving palliative intent treatment were excluded from study recruitment, though they still received shared‐care where clinically appropriate. Eligible patient participants were identified for recruitment at the start of HNC treatment and approached to gain informed consent. Consecutive recruitment was employed to reduce selection bias. The final participant cohort consisted of 23 individuals recruited across the 4 sites. Demographics are detailed in Table 1. As exploration of the demographics and representativeness of the eligible patient population is a key component of the ‘Reach’ domain, further discussion of the characteristics and generalisability of the patient population is detailed further in the results for Reach.

**TABLE 1:** Patient demographics, services/appointment details, and access costs (reported in Australian dollars, AUD) by site.

Patient demographics	Rural site 1	Rural site 2	Rural site 3	Remote site
	(*n* = 10)	(*n* = 1)	(*n* = 9)	(*n* = 3)
Male	8	1	7	3
Female	2	0	2	0
Mean age (range)	65.3 (43–79)	84	60 (55–73)	60.6 (55–65)
Employment status (paid)	6	0	4	3
Primary tumor site				
Oral cavity/oropharynx	6	0	6	3
Pharynx	1	0	1	0
Nasopharynx	1	0	0	0
Other (skin, parotid)	2	1	2	0
T‐classification				
0–2	4	0	4	2
3–4	6	1	4	1
Unknown	0	0	1	0
N‐classification				
0–1	6	0	4	2
2–3	4	0	4	1
Unknown/x	0	1	1	0
Treatment group				
RT only	1	0	0	0
CRT	5	0	5	3
Surgery + RT/CRT	4	1	4	0
Location of SLP services accessed				
Number of SLP sessions received				
At local site	39	1	25	16
At tertiary site	5	0	13	1
Travel distance and supports				
Attending with spouse/carer				
Local site	5	0	4	0
Tertiary site	9	0	8	2
Patient travel distance to local site				
Average	9.1 km	53 km	2.6 km	231.3 km
Range	1.3–13 km	—	0.6–6.8 km	1.9–569 km
Patient travel distance to tertiary site				
Average	92.8 km	165 km	130 km	862.6 km
Range	87–97 km	—	87–138 km	781–905 km
Patient costs associated with accessing service				
Patient reported costs per appointment ($AUD)				
To attend local site				
Average	$22.20	$10	$0.78	$375
Range	$0–$76	—	$0–$1	$0–$1080
To attend tertiary site				
Average	$427.30	$75	$290.06	$3968.33
Range	$28–$1320	—	$75–$945	$2825–$4550
Average difference in patient attributable costs for attending local vs. tertiary site appointments	$425.10	$65	$289.28	$3593.33

Abbreviations: CRT, chemoradiotherapy; km, kilometers; RT, radiotherapy.

Staff participants (*n* = 8) included speech‐language pathologists and SLP managers involved in implementing the shared‐care model across the 5 sites. These 8 staff members included 5 SLPs from the rural sites (1 each from rural site 1, 2, and 3; 2 from the remote site), and 3 from the tertiary site. The rural staff members had between < 1–24 years of SLP experience, < 1–7 years of HNC experience, and < 1–3 years of experience with the shared‐care model. The staff members from the tertiary service had between 2 and 35 years of SLP experience, < 1–20 years of HNC experience, and < 1–3 years of experience with the shared‐care model.

### 2.4 RE‐AIM Data Sources

The RE‐AIM evaluation was led by staff from the tertiary site who oversaw all aspects of data collection. Each RE‐AIM dimension was evaluated using a convergent parallel mixed‐methods approach, comparing and corroborating information from both qualitative and quantitative data sources to foster a rich and comprehensive understanding of implementation issues and overall service impact [34, 37]. Table 2 defines each RE‐AIM element and details the quantitative and qualitative data sources used to inform each RE‐AIM dimension.

**TABLE 2:** Dimension descriptions, and qualitative and quantitative data sources used for each RE‐AIM dimension.

Dimension and description^a^	Measures	Data sources
Reach *Number, proportion, and representativeness of individuals participating, and the reasons why/why not*	Quantitative ○ % and representativeness of participants ○ Reasons for non‐involvement ○ Patient demographics (participants, non‐participants, and target population) Qualitative ○ Staff perceptions of patient suitability	Service statistics Staff interviews
Effectiveness *The impact of the intervention on individual outcomes and broader impacts eg., quality of life and economic outcomes. Includes any negative effects and variability across subgroups*.	Quantitative ○ Number of sessions provided locally vs. non‐locally ○ Attrition rates ○ Quality of life (pre, post) ○ Costs saved in patient attributable travel costs Qualitative ○ Staff perceptions of effectiveness ○ Patient perceptions of effectiveness	Service statistics Quality of Life (EQ5D5L) Patient satisfaction survey Staff interviews Patient interviews Travel costs
Adoption *The absolute number, proportion, and representativeness of settings and intervention agents. Who is willing to initiate a program, and why?*	Quantitative ○ % and representativeness of settings and staff that participate Qualitative ○ Staff perceptions of adoptions	Service characteristics Staff interviews
Implementation *Fidelity in intervention delivery including time and costs involved. Any adaptations made to the intervention and implementation*.	Quantitative ○ Service statistics Qualitative ○ Implementation considerations from stakeholders ○ Staff perceptions of implementation ○ Patient perceptions of implementation	Service statistics Mid and End‐point stakeholder focus groups Staff interviews Patient interviews
Maintenance *(Setting level) ‐ the extent to which the intervention has become part of routine practice*	Qualitative ○ Staff and stakeholder perceptions of maintenance and sustainability	End‐point stakeholder focus group Staff interviews

^a^ Dimension descriptors as defined by Holtrop et al. [38].

#### 2.4.1 Quantitative Data Sources

Quantitative data sources included patient demographics, quality of life measures, travel costs, results from a patient satisfaction survey, and clinical service data. These were used to explore different aspects of the RE‐AIM framework as outlined in Table 2.

##### 2.4.1.1 Patient Demographics

Data pertaining to the patient's medical demographics were collected by the principal investigator (C.P.) from the electronic medical records.

##### 2.4.1.2 Quality of Life and Travel Costs Data

Patient participants provided quantitative data on their quality of life and travel costs at two timepoints: (1) Pre‐shared‐care: prior to the completion of oncology treatment at the tertiary site, and (2) Post‐shared‐care: either after 3 months of shared‐care (to reflect early post‐acute care) or at the time of resolution of issues requiring SLP services, whichever came first. Pre‐shared‐care, all patients completed the EQ‐5D‐5L, a standardized and validated measure of health‐related quality of life [39, 40]. The EQ‐5D‐5L contains (a) an assessment of five domains of health including mobility, self‐care, usual activities, pain/discomfort, and anxiety/depression rated on a 5‐point scale from none to extreme problems, and (b) a visual analogue scale (1–100) to rate overall health (from worst = 0 to best health you can imagine = 100). Participants also estimated costs for attending appointments at the tertiary centre, assuming all costs were self‐funded, including estimates of travel costs (plane tickets, fuel, time to travel), loss of income for themselves and any support person (income, leave entitlements), as well as associated expenses (childcare, meals, and accommodation) (see Supporting Information). Post‐shared‐care, patient participants repeated the EQ‐5D‐5L and provided cost estimates associated with attending SLP appointments at their local services. As self‐reported cost estimates may be subject to recall bias, these were collected during shared‐care to minimize retrospective distortion.

##### 2.4.1.3 Patient Satisfaction

Post shared‐care patient participants also completed a purpose‐built 5‐question satisfaction survey (see Supporting Information). This short survey explored their perceptions of the shared‐care model (e.g., *I was happy with the quality of care I received from my local speech pathologist*). Questions were rated on a 4‐point scale (strongly agree, agree, disagree, strongly disagree).

##### 2.4.1.4 Clinical Service Data

Quantitative data pertaining to the nature of each SLP session delivered were compiled by the treating clinician at the end of every session using a purpose‐built collection form (see Supporting Information). Data extracted from these forms included the number of appointments, the nature of appointments (e.g., clinical swallow assessment, instrumental swallow assessment, dysphagia rehabilitation, etc.), and service location (tertiary or local).

#### 2.4.2 Qualitative Data

Qualitative data sources included individual patient interviews, mid‐ and end‐project staff focus groups, and individual staff interviews. Interview design was consistent with the Qualitative Descriptive approach [41, 42]. Interview guides were developed with members of the research team (C.P., E.C.W., R.L.P., and J.F.) who have experience in qualitative methodology. Full details of the interview guides have been provided as Supporting Information. All interviews and focus groups were conducted via videoconferencing to support participation by the rural clinicians. All interviews were recorded, transcribed, and content checked for accuracy prior to analysis.

##### 2.4.2.1 Individual Patient Interviews

Patients were invited to complete an individual semi‐structured interview at the end of shared‐care to explore their experiences of the shared‐care model (e.g., “Is there anything you would change about the way you received your speech pathology services since you have returned home?”). A total of 19 of the 23 consenting patient participants completed an individual interview. Interviews were conducted via telehealth or phone by a member of the research team (R.L.P.), a mid‐career researcher and speech‐language pathologist with expertise in qualitative research and HNC. This researcher was not involved in any patient's clinical care to help mitigate participant reporting bias.

##### 2.4.2.2 Staff Focus Groups

Staff (*n* = 8) participated as a group in focus group interviews led by the principal study investigator (C.P.), held at (1) the study mid‐point, approximately 5 months post study commencement, and (2) the endpoint of the study period, once all patients had completed post assessments. The purpose of the focus groups was to review, as a group, the implementation of the shared‐care model and to share any issues regarding the ongoing implementation of the model. Key aspects of the model were considered and discussed (e.g., “Do you think the shared care model has been effective in supporting patients to access care post HNC treatment?”). At the end of the focus group, there was time allowed for an unstructured open‐ended forum to discuss any other issues regarding the models' implementation. Additional field notes collected by the lead investigator (C.P.) during informal contact with treating clinicians throughout the project were raised and discussed at this time, if relevant.

##### 2.4.2.3 Staff Individual Interviews

A single staff participant from each site (*n* = 5) was also invited to participate in individual, semi‐structured interviews to explore their perceptions of the model and its sustainability (e.g., “Can you describe benefits to your patients by using a shared model of care?”). This interview took place at the end of the study period. The individual staff interviews were conducted by an experienced qualitative researcher and speech‐language pathologist from the team (J.F.) who was external to the participating sites to minimize reporting bias.

### 2.5 Data Analysis

Data from qualitative and quantitative data sources were used to analyze each dimension (Table 2), informed by the RE‐AIM dimension descriptors [38] and consistent with prior research, which outlines data types typically reported within each RE‐AIM dimension [34]. One exception was the travel cost data. Costs are often discussed within the “Implementation” dimension of a RE‐AIM analysis [34]; however, in this study, reducing patient costs associated with accessing local care versus tertiary care was seen as an effectiveness measure for this model, and so cost data is analyzed under the “effectiveness” dimension in this study.

Descriptive statistics were calculated for all quantitative data variables (service data, patient costs, satisfaction questionnaires, EQ‐5D‐5L). In addition, to assess effectiveness, differences in EQ‐5D‐5L scores pre‐ and post‐shared‐care were analyzed using a Wilcoxon Signed Rank test for the five health domains, while a paired *t*‐test was used to analyze change in overall health state.

Costs for attending local and tertiary services, and the difference between both, were calculated and reported in Australian dollars (AUD). Total costs associated with accessing shared‐care for each patient (cost of attending all local appointments and any tertiary appointments) were calculated using the known costs, dates, and locations of SLP services provided. This data was compared with a simulated cost assuming all appointments were delivered only at the tertiary center (i.e., the historical model implemented before the introduction of shared‐care). The difference between these two costs served as an indication of the patient attributable costs that had been avoided by receiving shared‐care.

Qualitative data from patient interviews, staff interviews, and focus groups were analyzed by two study authors (C.P. and E.C.W.) using content analysis [43, 44]. Transcripts were independently reviewed and coded into categories and themes by two team members (C.P. and E.C.W.) in consultation with the researchers who conducted the interviews (R.L.P. for patient data, J.F. for staff data) to enhance analytical rigor. The interviewers then reviewed the final themes and categories to verify the interpretation, and discrepancies were resolved through discussion until consensus was reached. Once all qualitative data were analyzed, themes were reviewed and attributed to the relevant RE‐AIM dimension.

## 3 Results

### 3.1 Reach

As detailed in Table 2, the element of reach was examined using quantitative data from service statistics and qualitative data from staff interviews (Table 2). Synthesis of the quantitative and qualitative data revealed that all sources supported that the model had good reach and was addressing the needs of the intended target population.

#### 3.1.1 Service Statistics

During the 10‐month recruitment period, 34 rural patients received SLP services during their HNC treatment at the tertiary site. Of these, four were not considered for post‐treatment shared‐care as they had recently relocated from their rural home to live closer to the tertiary center. Two others did not require ongoing SLP services, and one patient declined further SLP care. Of the 27 rural patients who required ongoing SLP services post‐HNC treatment, only one had complex care requirements exceeding local SLP service capability and consequently remained with the tertiary site for management. The remaining 26 patients (96%) received SLP services via shared‐care on return home.

Of the 26 patients who received shared‐care, 23 (88.5%) consented and participated in the research evaluation (2 were ineligible due to palliative care status, 1 declined participation in the research). The demographic profile of the 23 consenting participants (Table 1) was broadly representative of the rural HNC population in the region; however, First Nations representation was lower than typical for rural sites (6% vs. the published rate of 14.6%) [45]. Of note, two patients who identified as First Nations received shared‐care during the period; however, they were ineligible for inclusion in the research due to their palliative status, as described above.

#### 3.1.2 Staff Interviews

Positive perceptions relating to the model's Reach were raised during staff interviews and coded under the theme “Patient Suitability” (Table 3). Clinicians felt the model was suitable for all patients and highlighted the ability for shared‐care to support patients from very remote communities. Benefits of the model for First Nations consumers were also recognized, with one staff member stating, “a lot of our cancer care patients identify as First Nations, and they definitely like to be ‘on country’. They feel more comfortable, they feel more at home, and you can tell that they are happier to be here” (S5).

**TABLE 3:** Themes and subthemes from staff interviews (*n* = 5) mapped to RE‐AIM domains.

RE‐AIM	Theme	Subtheme	Exemplar quote
Reach	Patient Suitability	First Nations Consumers	“I have done it with people who identify as First Nations… I think it's worked much the same as other patients” (S5)
		Suitable for all patients	[has it been difficult to provide shared care to any patient group] “No, I really don't think so!” (S4)
		Very remote communities	“We did have one under shared care who lived an hour and a half away [from rural site]… And we were able to do telehealth and that worked really well” (S5)
Effectiveness	Quality of care	Best practice	“Feels like good best practice.” (S4)
		Improve efficiencies	“It's probably just a more streamlined approach or service model … because of the processes and pathways that are in place.” (S4)
Adoption	Understanding benefits	Patient benefit	“It's quite expensive, it's an $800 return flight, it's a 10‐h drive… so, they definitely are happy to be home and in their own bed and comfortable.” (S5)
		Staff benefit	“So, this has allowed the rural clinicians to have a bit more of a deep delve into an area that perhaps traditionally wasn't offered.” (S4)
		Service benefit	“There's cost savings for the health service in terms of reducing need for patient travel [subsidy] applications.” (S4)
Implementation	Communication	Open communication	“It's a shared model… in terms of two‐way communication back and forth…Open communication makes it so much easier to facilitate the patients care, especially when they're complex and they do require to come back to [tertiary] for instrumental assessments, or when the doctors are asking questions about their progress.” (S1)
		Relationships	“It's been really good to feel comfortable, to pick up the phone and just be, hey, this person's coming. I know you know them, what do I do?…frequent communication between us and [tertiary] helps build that network and that connection, so we felt more comfortable” (S5)
		Support and development	“I think I didn't have a lot of confidence earlier on and I was reluctant to take on the oncology patients, but the support that's been provided has really helped me to not worry about it and to have that confidence.” (S2)
		Multidisciplinary collaboration	“There's been really good examples of where the speech pathologist is seeing them locally, it's enabled a joint session with [tertiary] dietitian via telehealth.” (S1)
Implementation	Infrastructure	Available resources	“The training that we did receive, I needed that, and all the written information that was there on file [in the resource repository] was really helpful.” (S2)
		Shared data systems	“Having the shared oncology dietician and speech notes available on EMR have been really good” (S3)
Maintenance	Alignment to strategy	Care closer to home	“One of our main pillars for our strategic plan and organization is to provide care closer to home and it's definitely been felt by our patients” (S5)
		Patient centred care	“I think it's a really great process because it's so patient centered and that's what we're always aiming for” (S3)
		Continuity of care	“It's a really good model from a continuity of care perspective” (S3)
		Workforce	“It's helped with our workplace staff upskilling, which is one of our pillars and in our strategic plan.” (S5)
Maintenance	Continuity of model	Business as usual	“When I rotated into this area, the shared care model was the norm. It's just standard care” (S1)
		Expansion to other caseloads	“It's a really good model, the way that it's laid out can be easily generalized to other caseloads…implementing with neuro rehab patients would be amazing” (S5)
		Challenges of rural staffing	“I have been through so many staff members of the speech team…it's a very transient workplace…if you last longer than a year, it's a miracle” (S5)

### 3.2 Effectiveness

Effectiveness was assessed through a convergent analysis of multiple quantitative data sources (service statistics, EQ‐5D‐5L, patient satisfaction survey, travel cost survey) and qualitative data (staff and patient interviews) (Table 2). Each of the quantitative data sources supported the effectiveness of the model in terms of the primary measure of effectiveness (providing SLP services closer to home) as well as secondary measures, including reduced travel costs, quality of life benefits, and overall satisfaction. Qualitative interviews with both patients and staff supported the benefits of the model.

#### 3.2.1 Service Statistics

The primary measure of effectiveness was the model's ability to enable eligible patients to receive SLP services close to home. Across the 100 SLP appointments provided to the 23 participating patients, the number of SLP sessions delivered locally was 4 times greater (*n* = 81, range = 1–10 sessions per patient) than those provided by the tertiary site (*n* = 19, range = 0–7 sessions per patient) (Table 1). All participants received at least some SLP care close to home, with 91% of patients receiving between 66% and 100% of SLP care locally (*n* = 16, 69.5%, received 100%; *n* = 5, 21.7%, received ≥ 66%). For the others, one patient received 50%, and one patient received 30% of their care locally. Reasons for this are discussed in the Implementation section. No patient withdrew from the shared‐care model.

#### 3.2.2 Travel Cost Data

Given that the model was designed to avoid travel to the tertiary center, the impact of the model on patient‐attributable costs was considered in the Effectiveness dimension. As shown in Table 1, the patient‐reported cost to attend an appointment at the local site (range = $0.78–$375) was considerably less than attending a session at the tertiary site (range = $75–$4550), with an average difference in patient‐attributable costs ranging from $65 to $3593.33 per appointment, depending on the location of the rural site. When applying these costs per appointment, and accounting for individual patient variation in proportion of rural versus tertiary appointments, the total patient‐attributable cost for attending the 3‐month episode/period of shared‐care (i.e., local appointments ± any tertiary appointments) on average was $891.89 (range = $0–$6710) per patient. In comparison, the ‘simulated’ cost per patient if all SLP care had been provided at the tertiary site (as per the historical care model) was on average $5100.63 (range = $35–$40 770). Thus, the difference in the patient‐attributable cost avoided by attending the shared‐care model was, on average, $4208.74 (range: $28–$40 365) per patient.

#### 3.2.3 Quality of Life

Although acknowledged as an indirect measure of model effectiveness, quality of life showed improvements at 3 months. Quality of life (EQ‐5D‐5L) data were available for 21 participants (2 had missing data). Pre‐post scores revealed an overall improvement of 12.38 points on the self‐rated health state, representing both a statistically significant improvement (pre: *M* = 56.19, SD = 17.31, post: *M* = 68.57, SD = 3.33, *t* = −3.140, *p* = 0.005) and a clinically meaningful change (i.e., exceeding the minimally important difference of 5 scale points [46]). Significant improvements were also observed across four of the five domains of health: mobility (pre: *M* = 1.71, SD = 0.85, post: *M* = 1.19, SD = 0.51, *T* = −2.49, *p* = 0.013), usual activities (pre: *M* = 2.95, SD = 1.53, post: *M* = 1.71, SD = 0.85, *T* = −3.124, *p* = 0.002), pain/discomfort (pre: *M* = 2.67, SD = 0.79, post: *M* = 1.67, SD = 0.79, *T* = −3.391, *p* ≤ 0.001), and anxiety/depression (pre: *M* = 2.09, SD = 1.14, post: *M* = 1.57, SD = 0.81, *T* = −2.428, *p* = 0.015). There was no significant change in self‐care, with minimal impacts reported pre or post (pre: *M* = 1.33, SD = 0.65, post: *M* = 1.05, SD = 0.22, *T* = −1.73, *p* = 0.08).

#### 3.2.4 Patient Satisfaction

Model effectiveness was also supported by the patient satisfaction survey findings. All patients (100%) reported that the service was beneficial, they were happy with the quality of care received, and the majority (91%) reported that they found it easier to access their SLP care locally. All patients saw the benefits of the model; they would recommend the service to other patients, and most (87%) participants were also happy to continue receiving all ongoing care locally.

#### 3.2.5 Patient Interviews

Consistent with the satisfaction survey results, the individual patient interviews revealed that patients viewed the model positively and spoke highly of the shared‐care service (see “Quality of Care” theme in Table 4). Specifically, many felt that the quality of care received from local and tertiary services was equivalent. Patients praised the attributes of clinicians in both services as both highly professional and personable, spoke of the ease of transitioning from the tertiary site to local care, and described the positive environment of the rural services. A second key theme relating to effectiveness was “Patient Benefits” (Table 4). Patients recognized multiple personal benefits from receiving local care. Attending local care required less personal effort, was less fatiguing, and reduced the burden on their family, who often assisted with travel. They commented that it was easier to access care locally, and it could be done as “just part of the day” (Pt26). In contrast, traveling to the tertiary site negatively affected employment, and the high personal burden and costs associated with this travel were a recurring concern (Table 4). Accessing care close to home was also perceived to have multiple benefits for well‐being and recovery, as well as a secondary benefit of ensuring the security of their home/property/pets.

**TABLE 4:** Themes and subthemes from patient interviews (*n* = 19) mapped to RE‐AIM domains.

RE‐AIM	Theme	Subtheme	Exemplar quote
Effectiveness	Quality Care	Equivalent care	It's, well, the exact same treatment I got it in [tertiary site], except it was only 5 min away. (Pt16) Everybody was fabulous. The service was terrific. I couldn't complain about nothing. (Pt26)
		Patient acceptability	When I was in having treatment, they sort of talked to me about [shared care] because I was living an hour away and there's someone [local] would I be interested, I said absolutely! (Pt5)
		Clinician attributes	The service itself is really good and they've been professional and empathetic (Pt17)
		Continuation of care	I have no problem at all. 'cause they reached out to me to start with. (Pt5)
		Environment	It was very, very easy to access, straight in and out there was no big waiting room where you joined a group of a dozen people waiting to be seen. (Pt12)
Effectiveness	Patient Benefits	Convenience	Having something local is a blessing, you know, so I don't have to do the whole trip to town (Pt5)
		Personal effort	After spending months driving up every day to [tertiary site] the last thing I want to do is drive [there] anymore. (Pt9)
		Fatigue	If I drive into [tertiary site] and back, it takes me 2 days to get over it…if you're just going down the street it's no impact on you (Pt26)
		Family	[Receiving care locally meant] I was still able to pick the kids up at the same time and things like that. (Pt16)
		Work	I was able to fit the appointment in around my work commitment. (Pt16)
		Costs	You're 6 h out of your day and $200.00 out of your pocket to come to [tertiary site]. (Pt12)
		Being Home	Being close [to home] is such an important part of your recovery (Pt26) To be honest, if we weren't in those circumstances, I probably would have bailed out because, you know, we got our dog and a lot of things here at the house. (Pt21)
Implementation	Operational elements	Communication across the pathway	Positive: I'm able to ring her anytime if I have any issues. (Pt5) Negative: I was told [by tertiary site] that the [local site] would organize appointments and more or less follow up exercises with me [but] there was times where I wouldn't hear from [local service] for eight weeks. (Pt12)
		Receiving care from two services	I have to come to [tertiary site] to do many other appointments so I could fit in the speech therapy but sometimes I didn't have to, so it was really good to have somebody local. (Pt5)
		Coordinating with the MDT	[Local speech pathologist] and the [tertiary site] dietitian actually consulted with me together [via telehealth]. (Pt7)
		Modality of care	It's been good, telehealth, I can get that done at the clinic…it was very flexible for me. (Pt3) [My local clinician] agreed to do home visits. So that was great. (Pt7)
		Local supports	You know if I pull it [NGT] out accidentally I could just pop down and she could have a look for me. Just the little things that make a huge difference. (Pt5)

Abbreviation: MDT, multidisciplinary team.

#### 3.2.6 Staff Interviews

Staff comments regarding the effectiveness of the shared‐care model were grouped under the theme “Quality of Care” (Table 3). Subthemes identified included the perception that the model enabled the delivery of best practice care. Clinicians also viewed it as a model that provided service efficiencies, as demonstrated by the exemplar quotes in Table 3.

### 3.3 Adoption

Insights into the Adoption of the model were drawn from quantitative analyses of service statistics and qualitative analyses of data from staff focus groups and individual interviews (Table 2). Overall, the multiple data sources supported the conclusion that the shared‐care model was successfully implemented and that all four rural sites were able to offer shared‐care. The local services involved were representative of the type of rural services and staff that this shared‐care model was designed for, that is, clinicians at the local sites had varying degrees of professional experience and prior HNC experience.

#### 3.3.1 Staff Interviews

Interviews with staff revealed that “Understanding the Benefits” (see Table 3) for patients was a significant influencing factor supporting the model's adoption. Benefits such as reduced travel burden and cost savings for patients and their families were frequently cited, as well as the convenience of traveling to their local center. Another subtheme was staff benefits, in particular, the opportunity to receive the training and support needed to upskill in SLP HNC clinical care, which was historically not offered to rural sites. Benefits to the service were a third subtheme, with staff noting the cost savings for the health service through reduced need for patient travel reimbursement (i.e., payments made by the public health service to alleviate patient costs).

#### 3.3.2 Staff Focus Groups

During the mid and end‐point stakeholder meetings, although staff were positive about the model, they noted that the adoption of the SLP HNC shared‐care model and delivery of local care were negatively influenced by episodes of staff turnover and staff leave. However, it was noted that during periods of staff leave/staff changeover at local sites, the tertiary service stepped back in to provide the ongoing care for patients (Table 5), which is a strength of the shared‐care model design.

**TABLE 5:** Implementation considerations from staff Mid‐ and End‐point staff focus group discussions (*n* = 8).

Considerations	Exemplar quote
Benefits/facilitators	
Prompt and clear communication	“[Effective shared patient care] always comes back to communication” (S1)
Clinicians working together to deliver patient care	“Nice for the patient to see that the two speech pathologists talk, are on the same page, and can go to either service depending on what's convenient. It's nice for the patient to know we are all invested in their care” (S1)
Early referrals to rural sites for preparation.	“Early referrals…helps me balance my calendar and my other patients. It helps me organize my time.” (S3)
Clear and detailed handovers	“It's a thorough handover that gives the key information…you can just pull it out on a busy day and just go” (S4)
Maintaining connection tertiary MDT members	“Linking in with the radiation oncologist through telehealth…if we've got any specific concerns—recurrence, pain management—that's impacting their oral intake” (S6)
Support and mentoring from tertiary clinicians	“Being a sole clinician, receiving support from an experienced clinician to provide oncology care…it's a supportive environment to gain experience and upskill” (S3)
Supporting patient care during periods of leave	“Talking about emergent and unplanned leave…I've been able to let [tertiary site] know…they provide timely care and the patient isn't waiting for me to come back” (S3)
Access to telehealth infrastructure	“It's great that we've got laptops that we can use for telehealth” (S2)
Local clinicians understanding local context	“Having the opportunity to link them to [local supports] is really good, and maybe something the [tertiary] team can't do…because I see them in their home setting” (S3)
Issues/barriers	
Breakdowns in communication	“If there's been barriers to communication between myself and rural sites, then it has been challenging to coordinate the patients care, especially if they're complex” (S1)
Limited uptake of telehealth handovers	“I have talked to the patient about how [telehandovers] would work…but they have declined [to participate]” (S1) “There's no time to do a telehandover…to organize it with rural clinicians because many are part time” (S1)
Site delays with EMR rollout	“With rurals going to electronic medical records this year, having access to notes will alleviate issues” (S4)
Knowing when to discharge.	“I'm not sure how long there's an expectation that we keep patients” (S4)

Abbreviations: EMR, electronic medical record; MDT, multidisciplinary team.

### 3.4 Implementation

Convergent analysis of the quantitative data from service statistics and qualitative data from staff focus groups, individual interviews, and patient interviews (Table 2) informed the analysis of the models' implementation. Overall, the data supported that the model has been successfully implemented. Rich data obtained from staff interviews, focus group discussions, and patient interviews highlighted elements that both support and can serve as barriers to the ongoing implementation of the model.

#### 3.4.1 Service Statistics

The fidelity of implementation was evaluated by reviewing the nature of sessions delivered by both the local and tertiary sites (i.e., whether appropriate services were delivered as per the shared‐care model). At the local sites, this encompassed services delivering routine SLP care, including subjective history taking (100%, *n* = 81), sourcing information from clinical information systems and documentation (100%, *n* = 81), oromotor assessments (44.4%, *n* = 36), informal speech assessment (4.9%, *n* = 4), informal voice assessments (2.5%, *n* = 2), clinical bedside swallow assessments (22.2%, *n* = 18), assessment of oral secretions (50.6%, *n* = 41), education and counseling regarding speech pathology intervention (95%, *n* = 77), oromotor therapy, that is, trismus management (28.4%, *n* = 23), communication therapy (8.6%, *n* = 7), dysphagia therapy (45.6%, *n* = 37), and education and training on how to manage secretions (71.6%, *n* = 58).

All SLP sessions provided by the tertiary site were deemed appropriate and consistent with the goals and intent of the shared‐care model: 8 sessions involved services only available from the tertiary site, including 7 videofluoroscopic swallow studies and 1 flexible endoscopic evaluation of swallowing; 7 were scheduled to align with other tertiary appointments for patient convenience, and 4 sessions were provided to cover local clinician leave. For the two patients who received 50% or less of their services locally, their local and tertiary appointments were reviewed and deemed to be consistent with the intent of shared‐care. One patient required multiple instrumental swallow assessments that could only be conducted at the tertiary facility. The other patient had care needs within the scope of the local clinician, as per the capability audit; however, their broader health care needs required regular multidisciplinary appointments at the tertiary service. Hence, as they regularly traveled to the tertiary site, SLP services were also provided there for patient convenience.

#### 3.4.2 Staff Focus Groups and Interviews

During both the staff interviews and the mid and end‐point stakeholder meetings, clinicians recognized the unique characteristics of the shared‐care model, along with enablers and challenges to implementation (Table 5). A key benefit emphasized by staff was that care was delivered collaboratively and that “it's not just simply a handover model” (S3). In particular, “Communication” (i.e., transparent, open, and timely interaction between sites; Tables 3 and 5) was viewed as a critical component of model success. Effective communication was perceived as supporting early initiation of referrals, while detailed clinical handover, “knowing when they're starting their treatment and expecting them to come back…you kind of feel like you know the patient before you meet them” (S5) helped to facilitate ongoing connection with the tertiary multi‐disciplinary team (MDT). The positive relationship between rural and tertiary sites was also considered central to ensuring the local clinicians felt supported and confident to provide care (Tables 3 and 5). “I've had the [tertiary] treating speech pathologist on telehealth while I've had the patient in the room to do shared‐care for a more complex patient” (S5).

A second key aspect of implementation success was “Infrastructure” (Table 3). Having shared clinical resources and a common EMR were advantageous for delivering the model. At the time of the study, some sites were awaiting EMR implementation. Once all local sites obtain full EMR functionality, it was recognized that there would be additional benefits for supporting shared‐care service delivery. Telehealth was also viewed as an important modality for supporting clinician–clinician communication and patient service delivery, particularly across extensive geographical distances. Although all clinicians had experience using telehealth for direct patient care, a key learning was the benefit of telehealth in supporting clinical handover and mentoring between clinicians +/− patient present (Table 5).

#### 3.4.3 Patient Interviews

Patients also recognized benefits in the way the model was delivered. Grouped under the theme of “Operational Elements” (Table 4), patients viewed open communication between their treating clinicians as critical in the shared‐care model. When breakdowns in communication occurred, these negatively impacted the patient experience (e.g., Pt12, Table 4). Knowing that they could seek services from either their local or tertiary clinician was viewed positively and offered patients flexibility in service access (Table 4). Even though they received their SLP care locally, patients viewed the joint telehealth sessions as a way of maintaining contact with the tertiary site.

### 3.5 Maintenance

Qualitative data from end‐point stakeholder focus groups and individual staff interviews were used to explore maintenance (Table 2). Since the shared‐care model had been running for 3 years, it was not surprising that staff felt the model was already well established and being sustained: “shared‐care is just business as usual for this particular caseload” (S4). They reported that having appropriate infrastructure and resources was essential, including telehealth systems, workplace policies and procedures, orientation manuals, and clinical handover documents (Tables 3 and 5). The model was also valued by staff. As shown by quotes in Table 3, they clearly articulated how the model demonstrated “Alignment to Strategy” and was consistent with health policy, providing patient‐centered care closer to home and ensuring care continuity following oncological treatment. Its ability to support workforce upskilling was also viewed as an advantage (Table 3).

Despite these benefits, retention of staff at rural sites was highlighted as an ongoing maintenance challenge for the model: “the staff turnaround is very frequent and transient. Most staff don't stay here for longer than 12 months.” (S5, Table 3). Staff were also keen to see the model expanded to other caseloads, such as neurorehabilitation, pediatric feeding, palliative care, cleft palate, and other specialized caseloads that are often not supported by rural generalist clinicians (Table 3). Some rural staff reported that they had already adapted concepts from the SLP HNC shared‐care model to other caseloads.

## 4 Discussion

This RE‐AIM implementation evaluation provides evidence supporting the efficacy and value of shared‐care in the delivery of post‐acute speech pathology services for HNC survivors in rural areas. Across a geographically dispersed health service, shared‐care enhanced rural workforce capability and allowed patients to access high‐quality, patient‐centred post‐treatment SLP care closer to home, while maintaining coordinated involvement with the tertiary centre. Three key findings emerged from this RE‐AIM evaluation, including: (1) shared‐care improved equity of access for SLP service for rural and remote patients while maintaining connections with specialist tertiary services, (2) the shared‐care model provided meaningful personal, financial, and psychosocial benefits to patients, and (3) shared‐care enhanced rural workforce capability through supported clinical practice and structured collaboration. The current findings demonstrate that shared‐care can be a feasible and valued service model within a rural HNC survivorship pathway, and can help to achieve the provision of care closer to home—aligning with key survivorship frameworks such as *Cancer Australia's National Framework for Cancer Survivorship* [47], the WHO's *Framework on Integrated People‐Centred Health Services* [48, 49] as well as advancing health‐equity priorities as outlined in *Queensland Health's Rural and Remote Health and Wellbeing Strategy* [49] and the *National Aboriginal and Torres Strait Islander Health Plan 2021–2031* [50].

### 4.1 Improved Equity of Access While Maintaining Tertiary Connections

A key outcome of this implementation evaluation was confirmation that the shared‐care model was able to overcome the challenge of distance and improve equity in access to HNC survivorship care for people living a significant geographic distance from their tertiary centers. All five participating sites adopted the model, which was underpinned by shared goals across the network, including a commitment to improving access to SLP care and a shared understanding of patient care developed through site engagement during model development [12, 13, 31]. All eligible rural patients received shared‐care, and almost all received at least two‐thirds of their appointments locally, demonstrating that the model successfully reached the target population and was effective in delivering care closer to home, overcoming distance as a known challenge to equitable HNC service access [4, 5, 51].

Importantly, this re‐distribution of care did not disconnect patients from tertiary support. Instead, the model preserved connections with the tertiary service for complex clinical needs (e.g., VFSS) and MDT services, as well as providing patient support during local leave/rural workforce turnover, which are common challenges in rural service models [52]. Connections between the rural and tertiary sites were supported through the use of a common EMR, routine case discussions, and telehealth support as needed, ensuring coordinated care [20, 25].

The model also accommodated individual patient needs. For some patients, care was delivered entirely by the local SLP service. However, for others, tertiary visits remained essential, or were necessary in case‐specific escalations, or during periods of unplanned or extended rural clinician leave. This flexibility in where services could be provided was identified as a strength of the shared‐care model. The dynamic, coordinated partnership between local and tertiary providers preserved access to quality SLP care, ensuring the individual needs of patients were met appropriately during the early post‐treatment recovery period.

Whilst the current shared‐care model was successful in improving equity of access, it is acknowledged that this was aided by the nature of the networked public health service in which this trial was conducted. Hence it may not be feasible to deliver this type of model within other healthcare systems, particularly where specialist cancer care teams have no formal connection to rural/local SLP services. In those contexts, finding ways to overcome issues relating to shared patient data, referral pathways, and managing reimbursement across the different services would provide particular challenges.

### 4.2 Meaningful Benefits to Patients

Patients reported high satisfaction with local SLP care, and described several meaningful logistical, financial, and psychosocial benefits of receiving support under a shared‐care model. Importantly, shared‐care was effective in supporting patients' psychosocial needs by keeping them in familiar settings, reducing disruption to employment and family responsibilities. These experiences likely influenced overall perceptions of wellbeing and may partially explain the observed improvements in quality of life indicators across the study period. This is consistent with other studies, which report that reduced travel burden and the ability to remain at home during recovery are critical enablers of wellbeing and continuity of care [3, 5, 19]. While these improvements cannot be attributed solely to the model—and may reflect natural recovery following treatment—the positive patient experience provides indirect evidence of model effectiveness.

It is recognized that significant financial toxicity is associated with cancer survivorship [5, 53, 54]. Hence, it is a positive finding that the model was effective in helping to avoid patient‐reported travel costs. Reduced travel, accommodation costs, and time away from work contributed to an estimated average total cost saving of $4008.74 per patient. These findings contribute to the growing body of evidence that decentralized shared‐care models can deliver both clinical and cost benefits for the patient [5, 6, 19].

### 4.3 Enhanced Rural Workforce Capability

In addition to direct patient benefits, the shared‐care model also enhanced rural workforce capability. All staff adopted the model and reported increased confidence and competence in managing HNC‐related swallowing and communication disorders. Although the Australian professional body, Speech Pathology Australia, suggests that all speech‐language pathologists should be able to manage non‐complex HNC cases [7], many rural clinicians report limited confidence due to infrequent clinical exposure and limited access to peer support [14]. Through structured mentoring and access to specialist input through shared‐care, rural clinicians working in this service model felt able to manage patients safely and effectively and work to full scope, enhancing job satisfaction and workforce skill mix—issues that have been closely linked to rural health retention strategies [23, 24]. Communication, which staff identified as the most significant factor influencing the successful implementation of shared care, was fostered by these structured and unstructured mechanisms for feedback, problem‐solving, and relationship building across sites [26]. The current data also confirm earlier study findings that suggest the model empowers the local workforce, promoting retention and long‐term system capability [30], which are essential elements in addressing rural workforce maldistribution [24].

Noting that the model evaluated in this study had been embedded for nearly 3 years, building clinician capacity and capability was recognized as central to the ongoing maintenance of the shared‐care model. Rural clinicians described the model as feasible and beneficial, though they acknowledged that sustained success depends on continued investment in training, supervision, and service infrastructure. This finding is consistent with the literature, which reports scalability of shared‐care models when supported by integrated systems, such as shared patient information, reliable telehealth infrastructure, and structured training [20, 25].

### 4.4 Broader Clinical and Service Delivery Implications

Beyond improved local access and patient experience gains, this evaluation also illustrates how distributed care can be operationalized at scale without fragmenting care. Pathways for maintaining good communication between the tertiary and rural sites were identified as the most critical factor influencing the successful implementation of shared care. Communication was optimized through coordination of early referrals, structured handovers, and ongoing informal mentoring and support. Reliable digital infrastructure, including a common EMR and telehealth capacity, was also a key facilitator in maintaining good communication between clinicians. Clear alignment with local health service policy was also viewed as a critical component supporting the ongoing maintenance of the service.

### 4.5 Limitations

This evaluation provides a valuable perspective on the implementation and outcomes of a shared‐care model; however, several limitations warrant emphasis. First, the evaluation window was limited, constraining the ability to assess long‐term sustainability and outcomes. Although the model had been in place for a number of years at the time of evaluation, ongoing monitoring and longitudinal data will be essential to determine whether the initial benefits of the model are maintained long‐term and to explore any changes to model design or new implementation challenges that arise over time. Also as previously discussed, the success of this model was aided by the networked public health context in which it was conducted, and as such other health services may be unable to replicate this form of coordinated care.

Additional data could also have been collected to enrich the analysis. Patient satisfaction and experience data were the primary measures used to determine the quality of care provided. Although patient perceptions offer meaningful insights into perceived benefit and acceptability, clinical indicators of quality and adherence to evidence‐based practice were not captured. Future work should incorporate clinical audits or standardized outcome measures in addition to patient‐reported outcome and experience measures. Where feasible, comparisons with historical or non‐shared‐care control cohorts could also be made.

It is also acknowledged that the economic estimates of avoided costs relied on assumptions about the number of tertiary appointments that would have occurred in the absence of shared‐care. Real‐world constraints, such as scheduling or travel budgets, may have resulted in lower tertiary appointments, potentially inflating estimated savings. Finally, although EQ‐5D‐5L scores improved and exceeded a recognized minimally important difference, these changes may reflect the natural recovery trajectory following treatment rather than a direct effect of the service model, and therefore, quality of life changes need to be considered with caution and are presented only as indirect markers of model effectiveness.

### 4.6 Conclusion

RE‐AIM analysis confirmed that the shared‐care model achieved the desired reach and was successfully adopted and implemented. Importantly, the model met its primary outcome, addressing inequities in post‐treatment SLP service access and delivering patient care closer to home. For patients, the model eased the burden of long‐distance travel, reduced time away from work and family, delivered care in familiar surroundings, and supported individual variability. It also ensured critically important tertiary site engagement was sustained, with local clinicians maintaining strong, coordinated links with the wider MDT and tertiary services, fostering collaboration across care settings. Key elements found to be central to the success of the model included clear communication, supported by a common EMR platform and telehealth services, as well as ongoing clinician training and support for local sites. Alignment with broader organizational goals on rural health equity and local health service policy was also central to ongoing service sustainability.

This evaluation demonstrates how targeted service redesign, informed by stakeholder consultation and grounded in implementation science, can address inequities in access and build capacity across rural SLP services. This model demonstrates a practical and scalable way to address long‐standing access issues for rural patients recovering from HNC treatment. With further refinement, this shared care model has the potential to extend to other SLP clinical caseloads (e.g., pediatric feeding, palliative care) and other allied health services, particularly those seeking to balance local care delivery for patients with easy and continued access to specialist input.

## Author Contributions

All authors contributed to the study conception and design. Recruitment of participants was facilitated by Corey Patterson and Sally Pether. Data collection and analysis were performed by Corey Patterson, Elizabeth Ward, Rebecca Packer, Jasmine Foley, and Laurelie R Wishart. The first draft of the manuscript was written by Corey Patterson and Elizabeth Ward, and all authors commented on subsequent versions of the manuscript. All authors read and approved the final manuscript.

## Funding

Project funding was provided by a Health Practitioner Research Grant from the Office of the Chief Allied Health Officer, Queensland Health.

## Ethics Statement

Ethical approval was sought and granted by The Townsville Hospital and Health Service Human Research Ethics Committee (HREC/18/QTHS/159).

## Consent

All staff and patient participants provided written informed consent.

## Conflicts of Interest

The authors declare no conflicts of interest.

## Supporting information


**Data S1:** hed70311‐sup‐0001‐Supinfo.docx.

## Data Availability

The data that support the findings of this study are available from the corresponding author upon reasonable request.
