# Supplementary material for: “It's the Exact Same Treatment … Except Only 5 Min Away”: A RE‐AIM Evaluation of a Speech‐Language Pathology Shared‐Care Model for Rural Patients With Head and Neck Cancer
Source: Head Neck. 2026 May 12;48(11):2977–94. doi: 10.1002/hed.70311 (PMC13653311; doi:10.1002/hed.70311)
Supplement: Supplementary file 1 [file HED-48-2977-s001.docx]

**Supplementary Material**

**Participant travel information and cost questionnaire**

Please answer the following questions about accessing speech language pathology services at

A. The tertiary site
B. Your local site

What is your?

1. Address
2. Closest local speech pathology service* (*Note: Asked only in “B” Local Site questionnaire)
3. Method of travel to appointments
4. Total transport costs for each return trip to attend a speech language pathology appointment (e.g.: car parking, flights, fuel)
5. Length of time to travel to local speech pathology appointment
6. Typical time spent at speech pathology appointment, including waiting and treatment time
7. Main activity if not attending an appointment (e.g.: work, looking after children, leisure activities)

Did you need to take time off paid work to attend the appointment?

Yes/No

If yes,

- How much time?
- How much would you lose in salary?
- What type of leave did you take?

Did anyone else attend the appointment with you (e.g. support person)?

Yes/No

If yes,

- Who attended with you?
- Did they need to take time off paid work?
- How much would they lose in salary?

Did you or your support person pay for child care in order to attend the appointment?

Yes/No

If yes,

a. How much was the child care?

What other costs might you and those attending with you incur (e.g.: meals, accommodation, other)?

**Patient Satisfaction Survey**

*Response: Strongly agree, Agree, Disagree, Strongly disagree, Unsure, Unable to comment*

1. I felt that receiving some of my speech pathology care closer to home was beneficial for me.
2. I found it was easier to access my speech pathology care when it was provided by my local therapist.
3. I was happy with the quality of care I received from my local speech pathologist.
4. I would prefer to receive all my speech pathology care from only the clinicians who treated me at [tertiary centre name].
5. I would recommend that future people with head and neck cancer receive care from local speech pathology services closer to their homes.

**Patient Interview guide**

1. Tell me about your experience of accessing speech pathology care closer to home? What was it like?
2. What did you like about accessing some of your speech pathology care closer to home?
3. Is there anything you would change about the way you received your speech pathology services since you have returned home?
4. Would you recommend to other patients that they receive part of their speech pathology care locally too? Why/ why not?
5. Is there anything else you would like to add, or tell me, about your experiences with this service?

**Staff Interview Guide**

1. What do you see are the drivers for you to support a shared model of care for patients with Head and Neck Cancer living in regional or remote areas?
2. Can you describe any benefits to your service through the use of a shared model of care?  *(prompt if only one answer given “Tell me about any other benefits?” or “Can you give me examples?”)*
3. Can you detail what training and supports you needed/required to be able to successfully provide care to patients post Head and Neck Cancer treatment using the shared-care model?  *(prompt “What was your skills in Head and Neck Cancer management prior to the model?  What is your experience using telehealth?  Have you ever provided a shared care model before?)*
4. Can you describe benefits to your patients by using a shared model of care? *(prompt if only one answer given “Tell me about any other benefits?” or “Can you give me examples?”)*
5. Tell me about any barriers you see in utilising a shared model of care. What changes/things you would like to be seen done differently moving forward? *(Thinking about the reach of this model, are there any patients or cohorts—such as First Nation’s/English as a second language/Very remote—who may not receive adequate care under the model?)*
6. Tell me about any facilitators that have helped you to utilise a shared model of care.  What things would you like to continue/build on moving forward?
7. What factors do you feel are required to sustain this shared care model into the future?  *(prompt if limited answer “What resources?” or “Any changes to the model?” or “Any local issues to consider?”)*
8. Do you think a shared model of care would work in any other patient populations?
9. Do you have any other thoughts or considerations you would like to add regarding your experiences with providing care to patients with Head and Neck Cancer using the shared care model?

**Staff Focus Group guide**

Reach

1. Do you think all of the people that could have benefited from the service received the service?
   a. If no, what made it difficult for them to access?  E.g, needing to travel to a tertiary facility for other appointments, limited local staffing, failed to attend appointments?
2. What do you think encouraged people to participate in shared care?
   a. For those who didn't want/couldn't participate, are there any strategies you think might work/did work?
3. What encouraged you, as a clinician, to participate in shared care?

Effectiveness

1. Do you think the shared care model has been effective in supporting patients to access care post HNC treatment?
2. Has the shared care model been effective in supporting you as a rural/remote clinician?
3. Has working in a shared care model helped you in any other ways?
   a. E.g.: Upskilling in another area of practice, building connections with MDT

Adoption

1. Is there anything that impacts you being able to utilise the shared care model?
   a. E.g.: staff leave, complexities of rural service provision, low numbers

Implementation

1. How has the model changed since you started using it (in 2019 vs more recently)?
2. Were there any unexpected adaptations to the model?
   a. E.g.: inclusion of MDT in shared care (dietetics)
3. We've already discussed a great deal about the barriers and facilitators to the model's implementation.  Is there any further feedback?

Maintenance

1. Were you using a shared care model prior to this evaluation?
2. How do you see the shared model of care aligning with the health service values and strategic pillars?
3. Do you think this model is sustainable long-term?
   a. If yes, what makes it sustainable?
   b. If not, why?  Is there anything that could be done to help make it sustainable?

**Patient Journey Form**

| **Enhancing Cancer Capabilities: Patient Journey** | |
| --- | --- |
| **Date of Session:** | **Patient UR:** |
| **Section 1. General info** | |
| IP/OP:  Modality: Telehealth/in person  Planned/unplanned:  Shared care/not shared care:  What was the goal of the session? (e.g.: assess reported dysphagia)  What clinical needs did the patient have? (e.g.: trismus impacting QoL)  What education, recommendations, or therapy did you provide?  Did you book a follow up appointment? If so, ?telehealth? | |
| **Section 2. Barriers and Facilitators** | |
| Where there any barriers to providing care to this patient (e.g.: unable to use telehealth, part-time therapist)?  Do you feel like the patient’s clinical needs were met? If no, why not? (e.g.: limited exposure or knowledge, time, patient didn’t understand)  Is there anything that would have supported you clinically to provide care to this patient?  Comments or additional info: | |
